# Overweight Is Associated With Medium‐Term Obesity Development in School‐Age Children: A Mixed Longitudinal Study

**DOI:** 10.1002/ajhb.70175

**Published:** 2025-11-30

**Authors:** Raphael Gustavo Testa, Adeluci Moraes, Aline Giselle Nagafuchi, Kamila Grandolfi, Andreo Fernando Aguiar, Diego Giulliano Destro Christofaro, Juliano Casonatto

**Affiliations:** ^1^ Research Group in Physiology and Physical Activity University Pitágoras UNOPAR Anhanguera Londrina Paraná Brazil; ^2^ Research Laboratory in Muscular System and Physical Exercise University Pitágoras UNOPAR Anhanguera Londrina Paraná Brazil; ^3^ Faculty of Science and Technology São Paulo State University (UNESP) ‐ Presidente Prudente São Paulo Brazil

**Keywords:** longitudinal study, nutritional status, obesity development, school‐age children

## Abstract

**Objectives:**

This study aims to investigate the dynamic changes in the nutritional status of school‐age children in five cohorts with a two‐year follow‐up, encompassing a span of 7 years.

**Methods:**

Utilizing a mixed longitudinal design, we implemented five cohorts with a two‐year follow‐up, encompassing a span of 7 years. The study's total sample comprised 101 school‐age children (51 females and 50 males), aged between 6 and 11 years at the commencement of the follow‐up. Anthropometric variables were obtained following standardized procedures. Subsequently, the Body Mass Index (BMI) was derived using the formula BMI = body mass (kg)/(height)^2^. Nutritional status was classified according to established cutoff points for age and sex.

**Results:**

The prevalence rates for overweight, obesity, and underweight were found to be 22%, 10%, and 5%, respectively. Notably, the presence of overweight at the initiation of the follow‐up exhibited a significant association with the development of obesity after a two‐year period (*χ* ^2^ = 5.325, *p* = 0.021). Furthermore, logistic regression analysis indicated that participants classified as “overweight” at the outset of the study were 4.7 times more likely (95% CI = 1.156–19.754) to develop obesity compared to their eutrophic counterparts.

**Conclusion:**

The study establishes a clear link between overweight status and the medium‐term development of obesity in school‐age children, aged between 6 and 11 years. These findings underscore the need for early intervention strategies and targeted preventive measures to address this concerning health issue.

## 1 Introduction

Childhood nutrition is widely recognized as a fundamental pillar of global public health and sustainable development efforts (Sotiraki et al. 2022). The nutritional status of school‐age children not only impacts their immediate health and well‐being but also has far‐reaching consequences for their cognitive development, academic performance, and future productivity (Asmare et al. 2018). As societies continue to evolve, driven by technological advancements, urbanization, and changing lifestyles, the nutritional landscape of school‐age children undergoes continuous shifts (Zhang et al. 2022). Consequently, there is an urgent need to examine and understand the evolving profile of changes in their nutritional status.

Despite substantial strides made by the global health community in addressing childhood malnutrition through various interventions, both undernutrition and overnutrition remain persistent challenges (FAO 2023). While some regions experience a decline in undernutrition rates, others grapple with escalating levels of childhood obesity and diet‐related non‐communicable diseases (Swinburn et al. 2019). The coexistence of these seemingly contradictory trends highlights the complexities of nutritional transitions among school‐age children and emphasizes the importance of a nuanced investigation into the drivers of these changes.

Socioeconomic status can influence obesity rates in high‐income school children (Turrell and Vandevijvere 2015). Paradoxically, affluence can be associated with higher obesity rates due to the greater availability of processed foods, sedentary entertainment options, and limited exposure to healthier lifestyles (Wu et al. 2015). With increasing urbanization and access to technology, high‐income Brazilian school children are leading more sedentary lifestyles (Corvalán et al. 2017). Extended screen time, including prolonged use of smartphones, computers, and video games, often replaces physical activities and outdoor play, contributing to an imbalance between energy intake and expenditure and promoting weight gain. Although our study did not directly measure physical activity or sedentary behavior, these factors are widely recognized as important determinants of childhood obesity and provide essential context for understanding the progression from overweight to obesity in this population (Haghjoo et al. 2022).

Given the potential long‐term health implications and public health significance, investigating the likelihood of overweight school‐age children developing obesity is of paramount importance. Understanding the factors contributing to the transition from overweight to obesity during childhood is critical for early intervention and prevention strategies. School‐age children who are already overweight may face compounding challenges in maintaining a healthy weight as they grow older, influenced by various physiological, environmental, and behavioral factors (Haghjoo et al. 2022). Identifying the determinants that drive this progression can inform targeted interventions and policies to halt the trajectory toward obesity and its associated comorbidities, such as cardiovascular diseases, type 2 diabetes, and musculoskeletal issues. Moreover, as obesity tends to track from childhood to adulthood, addressing the potential risk of overweight children developing obesity can have a lasting impact on reducing the burden of obesity‐related diseases in the population (Herman et al. 2009). Therefore, this study aims to describe and analyze the dynamic changes in the nutritional status of school‐age children across five cohorts with a two‐year follow‐up, spanning a total of 7 years, in order to identify the association between initial overweight status and the subsequent development of obesity.

## 2 Methods

### 2.1 Participants

**FIGURE 1:**
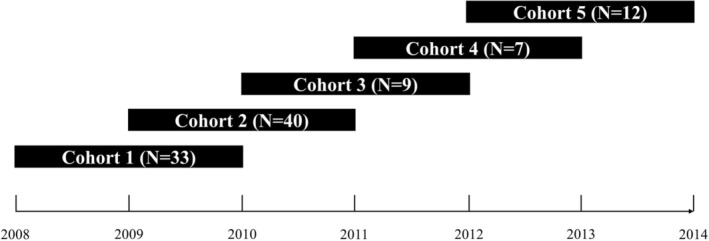
Study cohort design.

The present study adopts a mixed longitudinal cohort model, with two‐year follow‐up periods, initiated between 2008 and 2012 (Figure 1) in a private elementary and high school institution in the city of Londrina, Paraná, Brazil. Londrina is located 380 km from the state capital, Curitiba, and has an approximate population of 580,000 inhabitants, making it the second‐most populous city in the state and the fourth in the Southern Region. The city is an important urban, economic, industrial, financial, administrative, and cultural center in northern Paraná, with a Human Development Index (HDI) of 0.824.

Data for this study were obtained through an internal project aimed at monitoring anthropometric, habitual, and social indicators that may be related to the health status of the school community. The sample consisted of 101 students (51 females and 50 males) aged between 6 and 11 years at the commencement of the study, who were randomly selected from the student population of the institution. It is important to note that this educational institution caters to students from high and very high socio‐economic backgrounds (National Institute for Educational Studies and Research Anísio Teixeira (INEP) 2021).

All guardians of the students, after being informed about the purpose of this investigation and the procedures to be adopted, provided written informed consent. This study was conducted in accordance with the guidelines of Resolution 196/96 of the National Health Council for research involving human subjects, from the Ministry of Health, and was approved by the Research Ethics Committee of Universidade Norte do Paraná (CAAE No. 0281.0.268.000‐07).

### 2.2 Anthropometry

The participants' body mass was measured using a Filizola digital scale with a precision of 0.05 kg and a maximum capacity of 150 kg. Height was determined using a wooden stadiometer (Avanutri—AVA‐312) with a precision of 0.1 cm and a length of two meters, following the procedures described by Gordon et al. (Gordon et al. 1988), with the child standing without shoes, heels together, and head oriented with the Frankfurt plane. To determine body mass, the child was evaluated in the upright position, without shoes, and wearing a physical education uniform. The measurements of body mass and height were used to calculate the Body Mass Index (BMI) in kg/m^2^.

### 2.3 Nutritional Status

For the determination of the nutritional status, the cutoff points proposed by Cole et al. (Cole et al. 2000) for sex and age were adopted. According to these criteria, overweight and obesity were identified using the equivalent BMI curves of 25 and 30 kg/m^2^ for adults, respectively. Undernutrition was identified using the equivalent BMI curves of 18.5 and 17.5 kg/m^2^.

### 2.4 Data Analysis

Absolute and relative frequencies were used to present the prevalence data of undernutrition, normal weight, overweight, and obesity, both overall and stratified by sex. The distribution of the sample according to age at the beginning of the study was also presented. The distribution of the data was assessed using the Shapiro–Wilk test, and subsequently, the independent t‐test was applied to compare the general characteristics of each cohort between sexes.

The study employed the chi‐squared test to examine the relationship between the initial nutritional status (independent variable) and the occurrence of obesity (dependent variable) during the two‐year follow‐up period. Variables with *p* < 0.20 in bivariate analyses were entered into the logistic regression model to avoid prematurely excluding potential confounding variables, as recommended in epidemiological modeling approaches. This more inclusive threshold helps ensure that relevant predictors are not overlooked.

The binary logistic regression model was employed to assess the strength of the association between the initial nutritional status (independent variable) and the development of obesity (dependent variable), and was subsequently adjusted for sex to account for potential differences between boys and girls.

Descriptive statistics for numeric variables were presented as mean and standard deviation. The predetermined level of significance for all analyses was set at *p* < 0.05. All statistical analyses were conducted using SPSS 20.0 software.

## 3 Results

Table 1 presents the general characteristics of each cohort. When comparing between sexes, a remarkable similarity is observed in the variables age, height, weight, and BMI across all cohorts, with the only difference being in height. Specifically, in the 2008–2010 cohort, male participants had greater height, while in the 2009–2011 cohort, there was an inversion, with female participants showing higher scores in this variable. No other statistically significant differences were identified.

**TABLE 1:** Cohorts characteristics between male and female participants.^a^

Cohort 2008–2010										
	Male (*N* = 12)				Female (*N* = 16)				*t*	*p*
	Minimum	Maximum	Mean	SD	Minimum	Maximum	Mean	SD		
Age (years)	7	8	7.0	0.3	6	8	6.9	0.6	0.379	0.707
Height (cm)	117.0	128.6	122.5	3.6	111.6	131.6	119.7	5.7	2.082	0.046
Weight (kg)	19.4	34.0	25.9	3.9	17.6	33.3	25.2	4.3	1.870	0.071
BMI (kg/m^2^)	14.2	20.6	17.2	1.8	13.8	19.7	17.5	1.8	1.433	0.162

Cohort 2009–2011										
	Male (*N* = 20)				Female (*N* = 20)				*t*	*p*
	Minimum	Maximum	Mean	SD	Minimum	Maximum	Mean	SD		
Age (years)	7	9	7.2	0.5	7	8	7.4	0.5	−1.233	0.225
Height (cm)	110.0	133.1	121.8	6.3	114.8	135.5	126.0	5.3	−2.302	0.027
Weight (kg)	18.5	31.0	24.0	3.3	18.5	37.0	25.7	4.8	−1.341	0.188
BMI (kg/m^2^)	13.8	19.2	16.1	1.3	13.9	20.6	16.1	2.0	0.101	0.920

Cohort 2010–2012										
	Male (*N* = 4)				Female (*N* = 5)				*t*	*p*
	Minimum	Maximum	Mean	SD	Minimum	Maximum	Mean	SD		
Age (years)	8	10	9.0	0.8	8	9	8.4	0.5	1.323	0.227
Height (cm)	120.7	135.2	130.7	6.8	117.1	136.4	127.4	8.0	0.648	0.537
Weight (kg)	22.0	36.0	30.5	6.4	19.0	35.0	26.3	6.1	1.014	0.344
BMI (kg/m^2^)	15.1	20.2	17.7	2.6	13.9	18.8	16.0	1.9	1.148	0.289

Cohort 2011–2013										
	Male (*N* = 4)				Female (*N* = 2)				*t*	*p*
	Minimum	Maximum	Mean	SD	Minimum	Maximum	Mean	SD		
Age (years)	9	10	9.5	0.6	9	9	9.0	0.0	1.464	0.203
Height (cm)	137.8	141.7	140.0	1.7	127.3	132.4	129.8	3.6	1.751	0.140
Weight (kg)	29.2	37.8	34.0	4.0	23.3	25.8	24.5	1.8	0.122	0.908
BMI (kg/m^2^)	15.4	19.4	17.3	1.9	14.4	14.7	14.5	0.2	−0.243	0.817

Cohort 2012–2014										
	Male (*N* = 4)				Female (*N* = 4)					
	Minimum	Maximum	Mean	SD	Minimum	Maximum	Mean	SD	*t*	*p*
Age (years)	10	11	10.2	0.5	10	11	10.2	0.5	−0.310	0.763
Height (cm)	139.5	143.0	141.0	1.8	135.0	150.6	141.1	7.1	−0.395	0.701
Weight (kg)	36.6	42.3	39.3	2.4	30.1	49.6	36.4	8.9	0.410	0.690
BMI (kg/m^2^)	18.1	20.7	19.8	1.1	16.3	21.9	18.0	2.6	0.788	0.449

^a^ In this analysis, participants who started the follow‐up with obesity were not included.

Considering the age distribution (Table 2), 71% of the analyzed sample started the follow‐up between 7 and 8 years of age. Participants who began the study at 6 or 11 years of age accounted for 7% of the sample. This overall distribution was consistent with the distribution among sexes, with no significant discrepancy.

**TABLE 2:** Number of participants at the beginning of the follow‐up.

Age	Number of participants (*N*)	Percentage (%)	Number of participants (*N*)	Percentage (%)	Number of participants (*N*)	Percentage (%)
	Overall		Male		Female	
6 years	4	4.0	1	2.0	3	5.9
7 years	55	54.5	32	64.0	23	45.1
8 years	17	16.8	4	8.0	13	25.5
9 years	10	9.9	5	10.0	5	9.8
10 years	12	11.9	7	14.0	5	9.8
11 years	3	3.0	1	2.0	2	3.9
Total	101	100.0	50	100.0	51	100.0

The data regarding the nutritional status at the beginning of the follow‐up are presented in Table 3. The prevalence of “normal weight” was approximately 63%, while the prevalence of excess weight was 32%. Among this group, around 22% had overweight, and 10% were classified as obese. When considering the data stratified by sex, it was observed that the prevalence of “normal weight” was slightly higher among male participants (66% vs. 60.8%), as well as the prevalence of “obesity” (12% vs. 7.8%). On the other hand, the prevalence of “overweight” was higher in females (25.5% vs. 18%). As for “underweight,” the overall prevalence was 5%, with a slightly higher rate in females (5.9% vs. 4%).

**TABLE 3:** Distribution of participants' nutritional status by sex.

Nutritional status	Number of participants (*N*)	Percentage (%)	Number of participants (*N*)	Percentage (%)	Number of participants (*N*)	Percentage (%)
	General		Male		Female	
Underweight	5	5.0	2	4.0	3	5.9
Normal weight	64	63.4	33	66.0	31	60.8
Overweight	22	21.8	9	18.0	13	25.5
Obesity	10	9.9	6	12.0	4	7.8
Total	101	100.0	50	100.0	51	100.0

Table 4 presents the data analyzing the relationship between the participants' initial nutritional status, treated as the independent variable, and the occurrence of obesity development during the follow‐up. Initially, none of the participants with low weight became obese during the follow‐up, indicating the absence of an association between low initial weight and the risk of developing obesity. Among the 64 participants with normal weight at the beginning, the vast majority (93.8%) did not become obese during the follow‐up, while only four (6.2%) developed obesity, showing no statistically significant association. On the other hand, out of the 22 participants with overweight at the beginning, five (22.7%) developed obesity during the follow‐up, compared to only 5.8% of those who did not have overweight initially, revealing a statistically significant association (*p* = 0.021) between initial overweight and the risk of developing obesity during the follow‐up. It should be noted that, for the association analyses, participants who started the follow‐up with obesity were excluded.

**TABLE 4:** Association between nutritional status and development of obesity.^a^

		Became obese				*χ* ^2^	Phi	*p*
		No		Yes				
		*N*	%	*N*	%			
Underweight	No	77	93.8	9	100	0.581	−0.08	0.446
	Yes	5	6.1	0	0			
	Total	82	100	9	100			
Normal weight	No	22	26.8	5	55.6	3.207	−0.19	0.073
	Yes	60	73.2	4	44.4			
	Total	82	100	9	100			
Overweight	No	65	79.3	4	44.4	5.365	0.24	0.021
	Yes	17	20.7	5	55.6			
	Total	82	100	9	100			

^a^ In this analysis, participants who started the follow‐up with obesity were not included.

**FIGURE 2:**
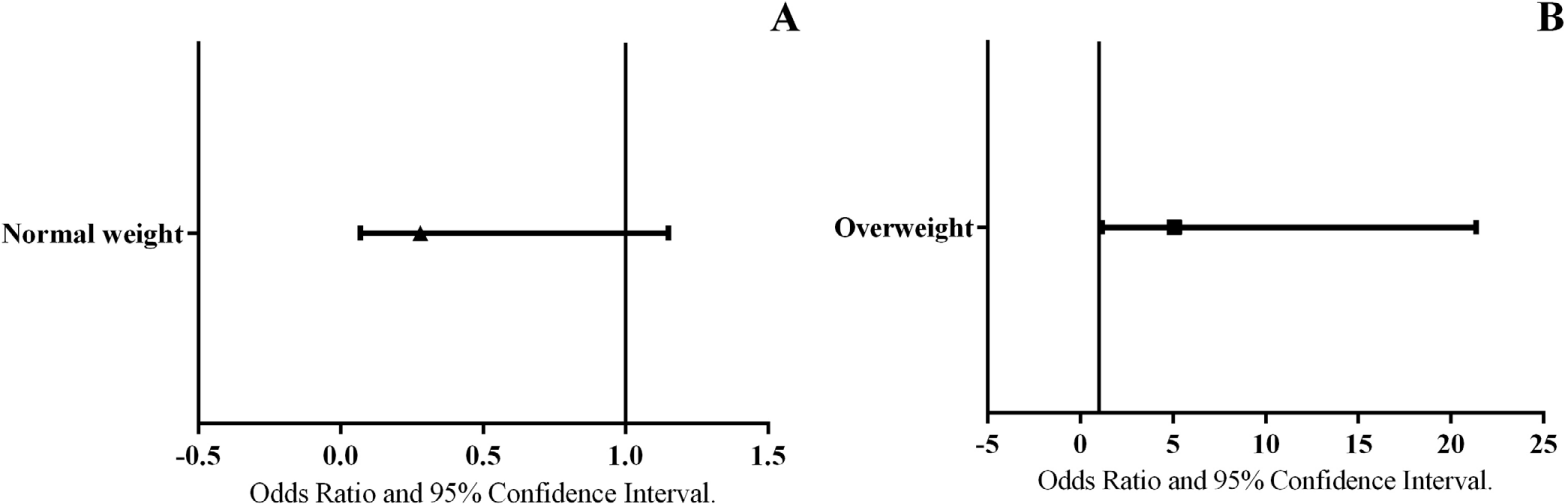
Magnitude of the association between baseline nutritional status—normal weight (A) and overweight (B)—and the development of obesity at follow‐up, adjusted for sex.

Figure 2 presents the data on the magnitude of the association between the independent variable (nutritional status at the beginning of the study) and the dependent variable (becoming obese at the end of the follow‐up period). There was no statistically significant odds ratio between normal weight at the beginning of the study and the development of obesity during the follow‐up (OR = 0.293–95% CI = 0.072–1.193). On the other hand, participants with overweight at the beginning of the study had 4.7 times higher odds of becoming obese during the follow‐up (OR = 4.779–95% CI = 1.156–19.754).

Figure 2 presents the data on the magnitude of the association between the independent variable (nutritional status at the beginning of the study) and the dependent variable (becoming obese at the end of the follow‐up period), adjusted for sex. There was no statistically significant association between being of normal weight at baseline and becoming obese during the follow‐up (adjusted OR = 0.28; 95% CI = 0.07–1.15; *p* = 0.076). In contrast, participants who were overweight at the beginning of the study had 5.1 times higher odds of becoming obese during the follow‐up (adjusted OR = 5.07; 95% CI = 1.20–21.37; *p* = 0.027). The variable sex was not a significant predictor in the model (*p* = 0.502 for the overweight model; *p* = 0.518 for the normal‐weight model).

## 4 Discussion

The main objective of this study was to examine the dynamic changes in the nutritional status of school‐age children, grouped into five follow‐up cohorts, over a total period of 6 years of investigation. The most notable finding from this research revealed that individuals with overweight at the beginning of the study had a 4.7 times higher likelihood of developing obesity in the subsequent 2 years, while those with normal weight did not show significant associations with the development of obesity.

Additionally, the analysis adjusted for sex showed that this variable did not significantly influence the associations between baseline nutritional status and the development of obesity. The magnitude and direction of the odds ratios remained virtually unchanged after adjustment, suggesting that, within this age range, the progression from overweight to obesity occurs similarly in boys and girls. This finding aligns with evidence indicating that, before puberty, sex‐related differences in body composition and metabolic regulation have limited impact on short‐term weight gain trajectories (Loomba‐Albrecht and Styne 2009).

It is important to characterize that overweight is a condition where there is an excessive accumulation of body weight relative to height, but still within the considered normal limits for age and sex (Nuttall 2015). It is a classification used to indicate a degree of excess weight that has not yet reached the stage of obesity (Nuttall 2015). Overweight can result from a variety of factors, including inadequate eating habits, lack of physical activity, genetic factors, underlying medical conditions, or a combination of several of these elements (Mazur et al. 2022).

On the other hand, obesity is a condition characterized by excessive accumulation of body fat, which can lead to significant health problems (Apovian 2016). In the pediatric population, obesity is a growing public health concern worldwide due to its negative repercussions on children's physical, psychological, and social well‐being (Rolland‐Cachera 2011). Childhood obesity results from an imbalance between caloric intake (food and beverages consumed) and caloric expenditure (physical activity and metabolism) (Bleich et al. 2011). Contributing factors to obesity include an inadequate diet, excessive consumption of calorie‐dense foods, lack of physical activity, sedentary behavior, genetic influence, obesogenic environment, and socioeconomic factors (Sahoo et al. 2015).

The results of this study emphasize the importance of understanding overweight as a health condition that deserves attention. Although overweight may initially seem relatively harmless, with few health implications, the results indicate that it becomes a significant risk factor for developing obesity in a short period. This finding is relevant, as it underscores the importance of not neglecting overweight since it can rapidly progress to a more serious and higher risk health condition.

This progression from overweight to obesity can be explained by various factors (Güngör 2014). First, the accumulation of body fat associated with overweight creates a favorable environment for metabolic and hormonal changes that facilitate additional weight gain (Han et al. 2010). Additionally, individuals in the intermediate zone between normal weight and obesity may already have some habits and behaviors contributing to weight gain, such as an unhealthy diet and lack of regular physical activity (Todd et al. 2015). These patterns can intensify over time, leading to a continuous cycle of weight gain and making it challenging to reverse the condition (Kumar and Kelly 2017).

A key takeaway from the study is the importance of preventing and effectively managing overweight. Early identification and addressing overweight can be crucial in preventing its progression to obesity, thereby reducing the risks associated with various chronic diseases such as type 2 diabetes (Ruze et al. 2023), cardiovascular diseases (Powell‐Wiley et al. 2021), and certain types of cancer (Scully et al. 2020). Preventive strategies, such as adopting healthy eating habits, increasing physical activity, and regular medical check‐ups, are essential in combating overweight and halting its evolution into obesity (Lee and Yoon 2018).

The present study showed that approximately 10% of the sample already had obesity, and 22% were overweight at the beginning of the follow‐up. These rates of obesity and overweight are in line with other data from Brazil (Bloch et al. 2016; Brazilian Ministry of Education 2018). However, some studies indicated even higher prevalence rates of obesity and overweight, such as 14% and 23% (Ghizzo Filho et al. 2021) and 15.9% and 15.4% (Panazzolo et al. 2013), respectively. When examining other studies reporting the prevalence of obesity and overweight, findings showed a variation in the obesity rate between 8% and approximately 18%, while overweight rates could reach around 25% (Cantanhede and Mariano 2020).

Additionally, it is noteworthy that the prevalence of overweight, as revealed in this study, was numerically higher among girls (25% vs. 18%), while the prevalence of obesity was higher among boys (12% vs. 8%). This gender‐related characteristic, i.e., a higher prevalence of overweight in girls and a higher prevalence of obesity in boys, has been identified in previous research (Costa et al. 2006).

Furthermore, it is essential to characterize that dynamic transformations in the nutritional status of school‐age children with high socioeconomic status may present some distinct peculiarities compared to other populations. Some of the key peculiarities include: (1) Access to food resources, as children from high socioeconomic status generally have access to a greater variety of foods and nutritional options (Alkerwi et al. 2015); (2) Obesogenic environment, as children from high socioeconomic status may be exposed to environments promoting sedentary behaviors and excessive consumption of calorie‐dense foods (Ozumut et al. 2020); (3) Lesser concern for food security, which may lead to greater consumption of processed and calorie‐dense foods (Valicente et al. 2023); (4) Influence of parental lifestyle, which can affect food choices and habits of their children (Mahmood et al. 2021); (5) Availability of physical activities, as children from high socioeconomic status may have more opportunities to engage in structured physical activities, such as organized sports, gyms, and clubs (Ferrari et al. 2020); and (6) Greater access to health information, as high socioeconomic status families tend to have more access to health and nutrition information, leading to more informed choices regarding food and lifestyle (Foroozanfar et al. 2022). It is important to emphasize that these peculiarities may vary depending on the context and specific characteristics of each population. Therefore, further studies are needed to investigate in detail the dynamic changes in the nutritional status of school‐age children with high socioeconomic status and their implications for health.

In conclusion, this study provides a comprehensive and insightful analysis of the prevalence of obesity and overweight among school‐age children over a significant follow‐up period. The identification of a significant relationship between initial nutritional status and the risk of developing obesity highlights the importance of early and effective interventions to prevent the progression to more serious health conditions. The results emphasize the need for preventive strategies and health promotion efforts specifically targeted at children with overweight to significantly reduce the impact of childhood obesity. Furthermore, the consistency between the prevalence rates found in this study and data from official sources of the Brazilian Ministry of Education (2018) enhances the reliability of the results obtained. Additionally, the adequate and representative sample size allows for a comprehensive view of the nutritional status of participants. Lastly, the study's longitudinal approach enables the analysis of trends and the identification of broader and more reliable associations.

However, it is important to acknowledge several limitations when interpreting the study's findings. First, the convenience sampling method used may not fully represent the study population, possibly introducing selection bias. Another important limitation of this study is the relatively small sample size and the fact that participants were recruited from a single private school, which primarily serves students from high and very high socioeconomic backgrounds. This characteristic limits the generalizability of our findings to broader populations, particularly those from different socioeconomic contexts.

The relatively short follow‐up period may also have limitations in capturing all changes in nutritional status over time. Unmeasured or inadequately controlled confounding factors may have influenced the associations found. Furthermore, the study focused on a specific region, which may limit the generalizability of the results to other populations and contexts. Therefore, it is necessary to interpret the findings with caution and consider these limitations when extrapolating the conclusions beyond the scope of this study. In addition, it should be recognized that obesity is a multifactorial condition, and the limited number of explanatory variables analyzed in this study does not allow for causal inference. Nevertheless, the use of a mixed longitudinal design, despite the sample restriction to a single private school, adds robustness by enabling the tracking of dynamic changes across multiple cohorts, strengthening the evidence that overweight is an important transitional state toward obesity.

## 5 Conclusion

The findings of this study indicate that overweight in school‐age children is statistically associated with an increased likelihood of developing obesity in the medium term. This observation reinforces the relevance of early identification and monitoring of overweight children within preventive health frameworks. However, given the multifactorial etiology of obesity and the limited explanatory variables analyzed, these results should be interpreted as preliminary and descriptive rather than deterministic. Broader studies with larger and more diverse samples, integrating behavioral, environmental, and biological markers, are necessary to elucidate the complex pathways that link childhood overweight to subsequent obesity.

## Author Contributions

R.G.T. and J.C. equally contributed to the conception and design of the research; A.F.A. and D.G.D.C. contributed to the design of the research; R.G.T., A.M., A.G.N., K.G. and J.C. contributed to the acquisition and analysis of the data; K. Grandolfi, A.F.A. and D.G.D.C. contributed to the interpretation of the data; and R.G.T. and J.C. drafted the manuscript. All authors critically revised the manuscript, agree to be fully accountable for ensuring the integrity and accuracy of the work, and read and approved the final manuscript.

## Funding

This study was supported by Fundação Nacional de Desenvolvimento do Ensino Superior Particular.

## Ethics Statement

All guardians of the participating students, after being informed about the purpose of the investigation and the procedures involved, provided written informed consent. The study complied with the guidelines of Resolution 196/96 of the National Health Council (Ministry of Health) for research involving human subjects and was approved by the Research Ethics Committee of Universidade Norte do Paraná (CAAE No. 0281.0.268.000‐07).

## Conflicts of Interest

The authors declare no conflicts of interest.

## Data Availability

The data that support the findings of this study are available from the corresponding author upon reasonable request.
